# Anoxic growth of *Ensifer meliloti* 1021 by N_2_O-reduction, a potential mitigation strategy

**DOI:** 10.3389/fmicb.2015.00537

**Published:** 2015-05-27

**Authors:** Emilio Bueno, Daniel Mania, Ǻsa Frostegard, Eulogio J. Bedmar, Lars R. Bakken, Maria J. Delgado

**Affiliations:** ^1^Department of Soil Microbiology and Symbiotic Systems, Estación Experimental del Zaidín, Spanish Council for Scientific ResearchGranada, Spain; ^2^Department of Environmental Sciences, Norwegian University of Life SciencesǺs, Norway; ^3^Department of Chemistry, Biotechnology and Food Science, Norwegian University of Life SciencesǺs, Norway

**Keywords:** denitrification, dinitrogen, greenhouse gas, nitric oxide, nitrous oxide reductase

## Abstract

Denitrification in agricultural soils is a major source of N_2_O. Legume crops enhance N_2_O emission by providing N-rich residues, thereby stimulating denitrification, both by free-living denitrifying bacteria and by the symbiont (rhizobium) within the nodules. However, there are limited data concerning N_2_O production and consumption by endosymbiotic bacteria associated with legume crops. It has been reported that the alfalfa endosymbiont *Ensifer meliloti* strain 1021, despite possessing and expressing the complete set of denitrification enzymes, is unable to grow via nitrate respiration under anoxic conditions. In the present study, we have demonstrated by using a robotized incubation system that this bacterium is able to grow through anaerobic respiration of N_2_O to N_2_. N_2_O reductase (N_2_OR) activity was not dependent on the presence of nitrogen oxyanions or NO, thus the expression could be induced by oxygen depletion alone. When incubated at pH 6, *E. meliloti* was unable to reduce N_2_O, corroborating previous observations found in both, extracted soil bacteria and *Paracoccus denitrificans* pure cultures, where expression of functional N_2_O reductase is difficult at low pH. Furthermore, the presence in the medium of highly reduced C-substrates, such as butyrate, negatively affected N_2_OR activity. The emission of N_2_O from soils can be lowered if legumes plants are inoculated with rhizobial strains overexpressing N_2_O reductase. This study demonstrates that strains like *E. meliloti* 1021, which do not produce N_2_O but are able to reduce the N_2_O emitted by other organisms, could act as even better N_2_O sinks.

## Introduction

The presence of N_2_O in the atmosphere has been known since 1939 (Adel, 1939). However, its importance to the global environment was not recognized until the early 1970s when scientists hypothesized that N_2_O released into the atmosphere could activate reactions in the stratosphere that contribute to the depletion of the ozone layer (Crutzen, 1974). The fourth assessment report of the intergovernmental Panel on Climate Change (IPCC, 2007) estimated N_2_O emissions from both natural and anthropogenic sources to be 8.5–27.7 Tg N_2_O/year. The terrestrial ecosystems are the main source of N_2_O, accounting about 65% of total emissions. Agricultural activities are the major sources of N_2_O emissions, accounting for 60–80% of the anthropogenic N_2_O sources, mostly as N inputs to agricultural soils (Smith, 2008; Smith et al., 2012). These N_2_O emissions are likely to increase with the predicted expansion in the use of nitrogenous fertilizers in order to satisfy the escalating demand for food of the growing world population.

A variety of biological pathways are involved in N_2_O emissions from soils, and it has been estimated that >65% of the atmospheric N_2_O derives from microbial N transformations, mainly through the processes nitrification and denitrification (Thomson et al., 2012). Of these, denitrification is generally considered to be the largest source of N_2_O and, depending on the type of microorganisms involved and the environmental conditions, this process can serve not only as source but also as sink for N_2_O (Thomson et al., 2012). Denitrification is the respiratory reduction of nitrogen oxides (NOx) which enables facultative aerobic bacteria to survive and multiply under oxygen-limiting conditions. During this process nitrate (NO^−^_3_) is converted into molecular nitrogen (N_2_) via nitrite (NO^−^_2_) and the gaseous intermediates nitric oxide (NO) and nitrous oxide (N_2_O) (Zumft, 1997).

In contrast to the variety of N_2_O sources in soils, removal of N_2_O is only achieved by the last step of the denitrification process which is catalyzed by the N_2_O reductase (N_2_OR) enzyme encoded by the *nosZ* gene. Recent reports have demonstrated that diverse microbial taxa possess divergent *nos* clusters with genes that are related yet evolutionarily distinct from the typical *nos* genes of denitrifiers (Sanford et al., 2012). In fact, phylogenetic analyses of the *nosZ* gene revealed two distinct clades of *nosZ* differing in their signal peptides, indicating differences in the translocation pathway to the N_2_OR across the membrane (Jones et al., 2013). The expression and activity of N_2_OR is a natural target in the search for options to mitigate N_2_O emission from agricultural soils (Richardson et al., 2009). A promising mitigation strategy suggested recently is to stimulate N_2_O reductase by sustaining a high soil pH (Bakken et al., 2012). The latter is motivated by recent demonstrations that reduction of N_2_O is severely inhibited by suboptimal pH in the model organism *Paracoccus denitrificans* (Bergaust et al., 2010), in bacterial communities extracted from soils (Liu et al., 2014), and in intact soils (Raut et al., 2012; Qu et al., 2014). Another interesting option would be to alter the composition of the denitrifying community of soils, the objective being to enhance the growth of organisms with high N_2_O reductase activity. This would be a daunting task if the free-living soil bacteria were the target, but plant-associated bacteria appear more promising.

Rhizobia is a general term that describes bacteria that have the ability to establish N_2_-fixing symbiosis in legume roots or on the stems of some aquatic leguminous plants. In addition to fixing N_2_, many rhizobial strains have genes for enzymes of some or all of the four reductase reactions for denitrification. Several studies have reported that legume crops induce N_2_O emission by providing N-rich residues for decomposition (Baggs et al., 2000). In addition to soil denitrifiers, endosymbiotic bacteria may be partly responsible for this legume-induced N_2_O emission, since most rhizobia are able to denitrify under free-living and under symbiotic conditions (Bedmar et al., 2005; Delgado et al., 2007; Sanchez et al., 2011). Increased N_2_O emissions due to degradation of nodules were reported in soybean ecosystems (Inaba et al., 2012). Based on this, Itakura et al. (2013) hypothesized and proved that N_2_O emission from soil could be reduced by inoculating soybean plants with a *nosZ*-overexpressing strain of *Bradyrhizobium japonicum*. This suggests that root nodules of leguminous plants are net sources or sinks for N_2_O. Thus, the investigation of denitrification among rhizobia may provide novel options for reducing N_2_O emissions from soils.

*Ensifer* (formerly *Sinorhizobium*) *meliloti* 1021 is a key model organism for studying the symbiotic interaction between rhizobia and plants of the genera *Medicago, Melilotus*, and *Trigonella*, that has also been extensively used in previous works to better understand the regulation and symbiotic characterisation of *E. meliloti* denitrification genes (Bobik et al., 2006; Meilhoc et al., 2010; Horchani et al., 2011). In fact, analysis of the *Ensifer meliloti* 1021 genome sequence revealed the presence of the *napEFDABC, nirK, norECBQD*, and *nosRZDFYLX* denitrification genes encoding a periplasmic nitrate reductase, a copper-containing nitrite reductase, a *c*-type nitric oxide reductase and a nitrous oxide reductase enzyme, respectively. The involvement of the *E. meliloti napA, nirK, norC*, and *nosZ* structural genes in nitrate respiration and in the expression of denitrification enzymes under specific growth conditions (initial oxygen concentrations of 2% and initial cell density of 0.2–0.25) was also demonstrated (Torres et al., 2014). However, this strain has for a long time been considered a partial denitrifier due to its apparent inability to grow under anaerobic conditions with nitrate or nitrite as final electron acceptors (Garcia-Plazaola et al., 1993; Torres et al., 2011a). In order to better understand the truncated denitrification phenotype of *E. meliloti* 1021, an accurate estimation of the efficiency of the denitrifying process is required. For that purpose, in this work we have used a robotized system which allowed us to simultaneously monitor the O_2_ consumption, as well as the consumption and production of each NOx during the transition from oxic to anoxic respiration.

The results convincingly demonstrated that this strain (1021) was unable to reduce NO^−^_3_ or NO^−^_2_ to N_2_O or N_2_. In contrast, this bacterium was capable to reduce externally supplied N_2_O to N_2_, serving as a terminal electron acceptor during anoxic respiration. Thus, our study expands the current understanding of anaerobic respiration in rhizobia and explores the effect of pH, NOx and type of carbon source on N_2_O reduction in *E. meliloti*.

## Materials and methods

### Bacterial strains, and growth conditions in batch cultures

*Ensifer meliloti* 1021 (Sm^r^, Meade et al., 1982), and *napA* (*napA*::mini-*Tn*5 Sm^r^, Km^r^, Pobigaylo et al., 2006) and *nirK* (*nirK*::mini-Tn5 Sm^r^, Km^r^, Pobigaylo et al., 2006) mutant strains were used in this study. *E. meliloti* strains were grown aerobically in 120 mL serum vials containing a triangular magnetic stirring bar and 50 mL of Triptone Yeast (TY) complete medium (Beringer, 1974) at 30°C. All cultures were continuously stirred at 700 rpm to avoid aggregation and ensure complete dispersal of cells. These cultures were then used as inocula into vials containing minimal defined medium (Robertsen et al., 1981) supplemented with or without 10 mM of KNO_3_ or 5 mM of NaNO_2_. The influence of carbon susbtrates on N_2_O uptake capacity was analyzed in minimal medium where the carbon substrate was replaced with either 5 mM of succinate or 5 mM of butyrate as oxidized or reduced carbon sources, respectively. The effect of pH on N_2_O uptake capacity was also studied in minimal medium strongly buffered (50 mM phosphate buffer) at pHs 6, 7, and 8. In all the treatments the headspace was filled with an initial concentration of O_2_ of 1 or 2% (12 or 24 μM dissolved O_2_ at 30°C, respectively). The headspace of experimental vials used to study the N_2_O reduction capacity was additionally supplied with an initial concentration of N_2_O of 2% (0.42 mM) or 5% (1.2 mM). To avoid possible external contaminations, antibiotics were added to the cultures at the following concentrations (μg mL^−1^); streptomycin, 200; kanamycin, 200.

### Preparation of incubation vials

120 mL vials containing 50 mL liquid medium were crimp-sealed with rubber septa (Matriks AS, Norway) and aluminum caps to ensure an airtight system. Oxygen from vials was removed by 6 cycles of air evacuation during 360 s and helium (He) filling during 40 s. Constant stirring (400 rpm) was kept to ensure optimal gas exchange between liquid and headspace. Then, vials were injected with the required concentrations of O_2_ and N_2_O.

### Gas measurements

After inoculation, cultures, blanks, and gas standards were placed in a thermostatic water incubator containing a serial magnetic stirrer at 30°C, with continuous stirring at 700 rpm, and the gas kinetics were monitored in each vial (2 to 3 h intervals). The gas measurements were performed by monitoring the headspace-concentrations of relevant gases (O_2_, CO_2_, NO, N_2_O, and N_2_) by repeated gas sampling through the rubber septa of the incubation vials as described by Molstad et al. (2007). The gas samples were drawn by a peristaltic pump coupled to an autosampler (Agilent GC Sampler 80), and with each sampling an equal volume of He was pumped back into the vials. This secured that the gas pressure was sustained near 1 atm despite repeated sampling, but diluted the headspace atmosphere (with He). This dilution was taken into account when calculating rates of production/consumption for each time increment (Molstad et al., 2007). The sampling system was coupled to a gas chromatograph (GC) (Agilent GC -7890A) with two 30 m × 0.53 mm id columns: a Porous Layer Open Tubular (PLOT) column for separation of CH_4_, CO_2_ and N_2_O, and a Molsieve column for separation of O_2_ and N_2_ (and Ar, Ne). The GC had three detectors: a flame ionization detector (FID), a thermal conductivity detector (TCD), and an electron capture detector (ECD). N_2_O was detected by both the ECD and TCD, thus securing accurate measurements at near-ambient concentrations (ECD, linear range 0–4 or 0–20 ppmv, depending on detector temperature) and linear response for higher concentrations (TCD). NO concentrations were determined by a Chemoluminiscence NOx analyser (Model 200A, Advanced Pollution Instrumentation, San Diego, USA).

### OD_600_, nitrate and nitrite measurements

Cell densities (OD_600_), nitrate and nitrite concentrations were measured for each sample. Samples were taken from the liquid phase of the vials throughout the experiment to measure OD_600_ (0.7 mL sample), NO^−^_3_(0.1 mL sample), and NO^−^_2_ (0.1 mL sample) using sterile syringes. For determination of NO^−^_3_, a 10 μL aliquot was injected into a purge vessel with heating jacket and condenser (ASM 03292) containing 1 M HCl and vanadium (III) chloride. Temperature of vessel was controlled by a circulating water bath at 95°C and cold water for the condenser. In addition, a gas bubble/NaOH trap with Teflon sleeve (ASM 04000) was used to avoid the corrosive effects of HCl. Vanadium (III)/HCl converts nitrite and S-nitrosocompounds to NO, which is transported (by N_2_) to a chemiluminescence detector Nitric Oxide Analyzer NOA 280i (General Electric). N_2_ was continuously bubbled through the reducing agent to maintain an anaerobic environment in the system and to transport the NO through the NO analyzer (Walters et al., 1987). The approximate detection limit was 1 pmol NO, equivalent to 0.1 μM (when injecting 10 μL). For determination of NO^−^_2_, a 10 μL subsample was injected into a purge vessel (gas bubble/NaOH trap is not needed) containing acetic acid with 1% vol NaI where NO^−^_2_ is converted to NO.

### Analyses of kinetics of aerobic and anoxic NO^−^_3_, NO^−^_2_, or N_2_O respiration

Experimental dataset obtained from the series of incubations were used to determine the kinetics of O_2_, NO^−^_3_ NO^−^_2_, or N_2_O respiration and NO, N_2_O, and N_2_ production in order to provide the most accurate information on *E. meliloti* physiology during the transition from aerobic to NOx anoxic respiration. O_2_ and NO concentration in the liquid, determined as μM and nM, respectively, was estimated taking into account the partial pressure of these gases at headspace, their solubilities and transport coefficients between headspace and liquid. Additionally, O_2_ concentration in liquid was estimated respective the O_2_ respiration rate for each time increment (see Molstad et al., 2007 for details). N_2_O was analyzed as μmol N_2_O vial^−1^, whereas N_2_ was determined as cumulative net production of N_2_. All data were corrected for dilution rates and losses by gas sampling, and leaks due to gas diffusion through the rubber septa. The concentrations of NO^−^_3_ and NO^−^_2_ were determined at different times compared to the gas sampling. However, we needed values for NO^−^_2_ concentrations at the same time as the gas sampling in order to estimate electron flow rates. For this reason, polynomial functions [f(t)] were fitted to the measured NO^−^_3_ and NO^−^_2_ concentrations, and used to estimate NO^−^_2_ concentration at the time of gas samplings. Graphical presentations for NO^−^_3_ and NO^−^_2_ concentrations include both measured data points and the polynomial function.

The apparent growth rates based on O_2_ consumption (μ_ox_), and reduction of any NOx during the anoxic phase (μ_anox_) were estimated by regression [ln (V_e−_) against time] for the phases with exponentially increasing rates. Yield (cells pmol^−1^ e^−^) calculation was based on the number of cells rendered per pmol electron used by the respiratory terminal oxidases to reduce O_2_ to H_2_O during oxic phase (Yield_ox_) or by the complete set of denitrifying reductases to reduce NO^−^_3_ NO^−^_2_ or N_2_O to N_2_ during anoxic phase (Yield_anox_). V_max_ is an useful parameter that can tell us the efficiency for O_2_ and NOx respiration per cell. It estimates the maximal velocities per cell and per hour for the reduction of O_2_ and NO_x_. This parameter is based on the fmol of electrons used by the terminal oxidases and denitrifying enzymes to reduce O_2_ or NOx, respectively, per cell and per hour. For further details regarding these calculations, see Molstad et al. (2007) and Nadeem et al. (2013).

## Results

### Kinetics of aerobic respiration

**Figure 1:**
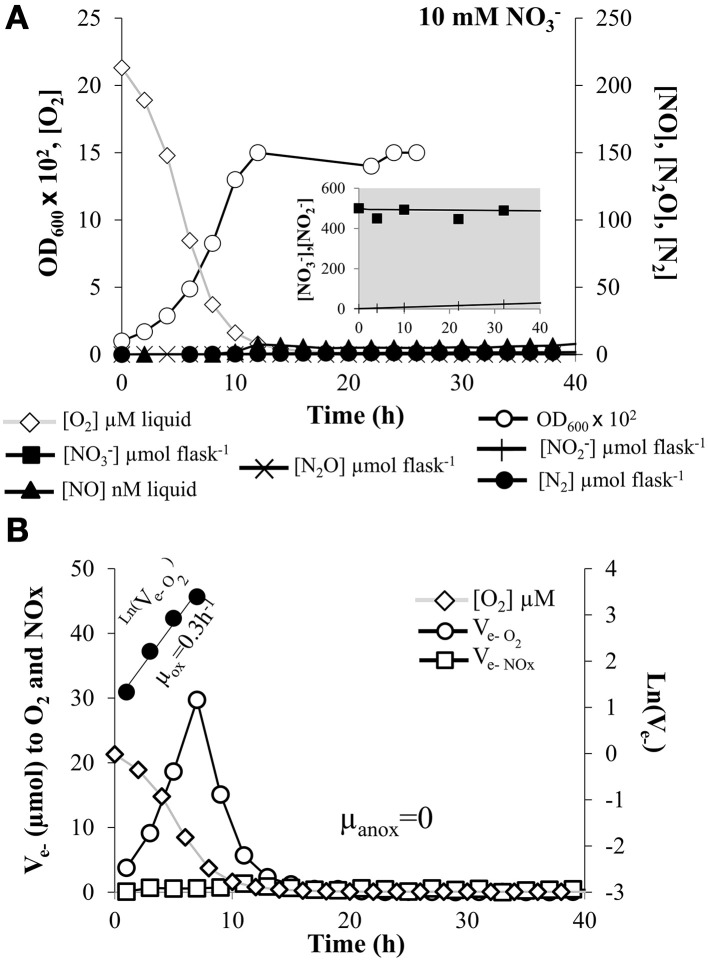
**Kinetics of O_2_ and NO^−^_3_ respiration. (A)** Absorbance at 600 nm (OD_600_), O_2_ consumption, NO^−^_3_ depletion (insert), and production of NO^−^_2_ (insert), NO, N_2_O, and N_2_ by *E. meliloti* 1021 when incubated in the presence of 10 mM NO^−^_3_ in the medium and an initial O_2_ concentration of 2% in the headspace. **(B)** The electron flow rate to O_2_ is shown as log-transformed values for the phases with exponential increase (filled circle symbols). The slopes estimating apparent growth rates were 0.3 (± 0.03) h^−1^ and 0 for oxic and anoxic phase, respectively. Cultures with an initial OD_600_ of 0.01 were vigorously stirred at 700 rpm. The result shown is for a single vial. Several replicates were analyzed, with similar results, although the exact timing of events was not the same. However, the consistency of the observations is demonstrated in Table 1 where averages of at least three different cultures are reported.

**Figure 2:**
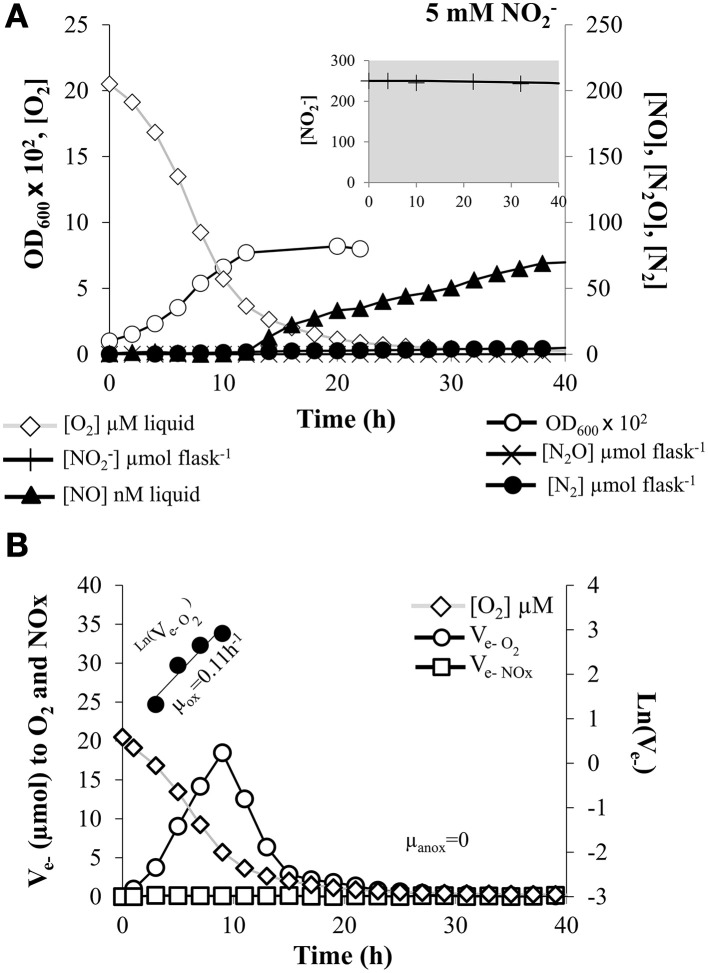
**Kinetics of O_2_ and NO^−^_2_ respiration. (A)** Absorbance at 600 nm (OD_600_), O_2_ consumption, NO^−^_2_ depletion (insert), and production of NO, N_2_O, and N_2_ by *E. meliloti* 1021 when incubated in the presence of 5 mM NO^−^_2_ in the medium and an initial O_2_ concentration of2% in the headspace. **(B)** The electron flow to O_2_ shown as log-transformed values for the phases with exponential increase (filled circle symbols). The slopes estimating apparent growth rates were 0.11 (±0.02) h^−1^ and 0 for oxic and anoxic phase, respectively. Cultures with an initial OD_600_ of 0.01 were vigorously stirred at 700 rpm. The result shown is for a single vial. Several replicates were analyzed, with similar results, although the exact timing of events was not the same. However, the consistency of the observations is demonstrated in Table 1 where averages of at least three different cultures are reported.

**Figure 3:**
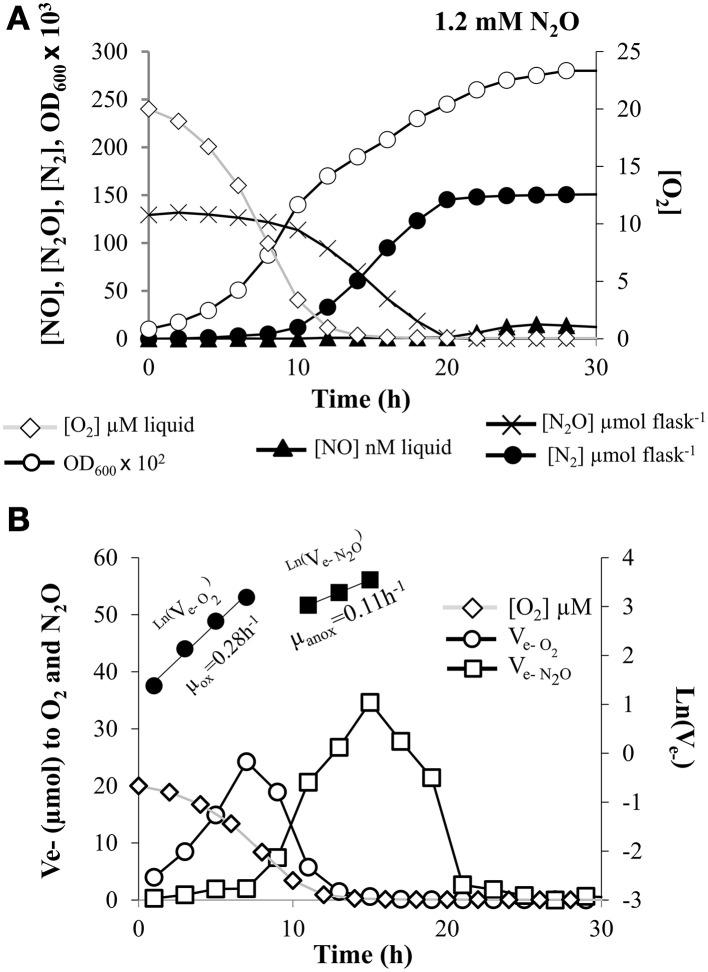
**Kinetics of O_2_ and N_2_O respiration. (A)** Absorbance at 600 nm (OD_600_), O_2_ and N_2_O depletion, and production of NO, and N_2_ by *E*. *meliloti* 1021 when incubated in the presence of 10 mM NO^−^_3_ in the medium and an initial O_2_ concentration of 2% in the headspace plus 5% N_2_O. **(B)** The electron flow to O_2_ or to N_2_O is shown as log-transformed values for the oxic (filled circle symbols) and anoxic phase (filled square symbols) with exponential increase. The slopes estimating apparent growth rates were 0.28 (±0.03) h^−1^ for the oxic phase, and 0.11 (±0.03) h^−1^ for the anoxic phase, respectively. Cultures with an initial OD_600_ of 0.01 were vigorously stirred at 700 rpm. The result shown is for a single vial. Three replicates were analyzed in parallel with similar results. Consistency of the observations are demonstrated in Table 1 where averages of at least three different cultures are reported.

*E. meliloti* strain 1021 was grown aerobically for 30 h with vigorous stirring (700 rpm) until a maximal optical density at 600 nm (OD_600_) of ~0.3 to avoid generation of localized anoxic conditions due to cell aggregation. Then, an aliquot was used to inoculate the culture vials to an initial OD_600_ of 0.01 (8 × 10^6^ cells mL^−1^). The medium contained either 10 mM of nitrate (Figure 1), 5 mM NO^−^_2_ (Figure 2) or 10 mM nitrate plus 5% N_2_O (1.2 mM N_2_O concentration in the liquid when in equilibrium with the headspace) (Figure 3). In all the treatments for studying the kinetics of aerobic respiration, the initial O_2_ concentration in the headspace was 2%. Figure 1A shows the measured OD_600_, O_2_, NO, N_2_O, and N_2_ concentrations in the medium for a single vial throughout the 40 h incubation in the presence of nitrate. NO^−^_3_ depletion and production of NO^−^_2_ is also shown (Figure 1A, insert). In nitrate-treated cells, oxygen was consumed within the first 15 h, OD_600_ increased linearly with the cumulative O_2_ consumption (*r*^2^) = 0.9877, and remained practically constant throughout the anoxic phase. Rates of O_2_ consumption for each time increment between two samplings were used to calculate electron (e^−^) flow rates to oxygen (V_e−O2_). As shown in Figure 1B, V_e−O2_ increased exponentially throughout the first 7 h in proportion with the increase in OD_600_ (*r*^2^ = 0.9105), and declined gradually in response to diminishing O_2_ concentrations. The initial exponential increase in electron flow during oxic respiration can be taken as an indirect measure of growth rate (μ_ox_) (Liu et al., 2013). Thus, the apparent μ_ox_ estimated by linear regression of ln (V_e−O2_) against time was 0.30 (±0.03) h^−1^ (Figure 1B, Table 1A). The final OD_600_ was 0.15 (±0.02) (1.60 × 10^8^ cells mL^−1^, Table 1B) resulting in a yield of 24.6 (±2.8) cells pmol^−1^e^−^ to O_2_ (Table 1A). The apparent maximum specific respiration rate, V_max_, which is a useful indicator of the respiration per cell, was 11.6 (±0.5) fmol e^−^ cell^−1^ h^−1^ for oxygen respiration in cells grown in the presence of nitrate (Table 1A).

**Table 1:** **Summary of oxic and anoxic growth parameters **(A)**^1^ Depending on the presence of nitrogen oxides, and the subsequent conversion of the nitrogen oxides present **(B)**^2^**.

**(A)**						
	**Growth parameters for the oxic phase**			**Growth parameters for the anoxic phase**		
**NO_x_ present (mM)**	**μ_ox_ (h^-1^)**	**Yield_ox_ (cell pmol^-1^ e-)**	**V_max_ (fmol e- cell^-1^ h^-1^)**	**μ_anox_ (h^−1^)**	**Yield_anox_ (cell pmol^-1^ e-)**	**V_max_ (fmol e- cell^-1^ h^-1^)**
NO^−^_3_(10)	0.30(±0.03) a	24.6(±2.8) a	11.6(±0.5) a	0	0	0
NO^−^_2_(5)	0.11(±0.02) b	14.1(±1.1) b	8.2(±0.7) b	0	0	0
N_2_O(1.2)^3^	0.28(±0.03)a	23.1(±6.2) a	8.9(±0.1) b	0.11(±0.03)	18(±0.6)	5.7(±1.1)
**(B)**						
**NO_x_ present (mM)**	**[O_2_] at onset of NO_x-_reduction (μMO_2_)**		**Max [NO−] in liquid (nM NO)**		**Final OD (OD_600_)**	
NO^−^_3_(10)	2.7(±1.5) a		12.4(±2.10) a		0.90(±0.30) a	0.15(±0.02) a
NO^−^_2_(5)	3.0(±0.7) a		94.2(±16.9) b		0.18(±0.02) b	0.08(±0.01) b
N_2_O(1.2)^3^	5.9(±2.6) b		15.0(±1.10) a		100(±2.50) c	0.28(±0.05) c

*The alternative respiratory substrate (NOx) present in the medium (NO^−^_3_ or NO^−^_2_) or at headspace (N_2_O) for each analysis is indicated. All the experimental vials contained an initial O_2_ concentration of 2% at headspace. Data are means with standard error (in parenthesis) from at least three independent cultures. Values in a column followed by the same lower-case letter are not significantly different according to One-Way ANOVA and the Tukey HSD test at P ≤ 0.05*.

*^1^Apparent oxic growth (μ_ox_, h^−1^) and anoxic growth (μ_anox_, h^−1^) rates based on O_2_ consumption during the oxic phase or reduction of NO^−^_3_, NO^−^_2_, or N_2_O during the anoxic phase. Yield (cells per mole electron) based on increase in OD vs. cumulated consumption of oxygen or reduction of NO^−^_3_, NO^−^_2_, or N_2_O, and apparent maximum specific respiration rate (V_max_, fmol electrons cell^−1^ h^−1^) during the initial phase (0–5 h) of the experiments (Figures 1, 2)*.

*^2^The oxygen concentration at the time of the first indications of anoxic respiration (i.e., appearance of NO in the treatments with NO^−^_3_ and NO^−^_2_, and appearance of significant N_2_O reduction to N_2_ in the treatment with N_2_O)*.

*^3^5 % N_2_O (150 μmol N_2_O at 20°C) was injected into each vial, resulting in 1.1 mM N_2_O in the liquid when in equilibrium with the headspace*.

O_2_ uptake and growth kinetics were also analyzed in cells grown in the presence of 5 mM NO^−^_2_ as final electron acceptor (Figure 2). For this treatment, O_2_ was consumed within the first 30 h of incubation showing a delay in comparison to NO^−^_3_ treatment (Figure 2A). As observed in nitrate-treated cells, OD_600_ also increased during the oxic phase in proportion with O_2_ consumption, and remained constant during the anoxic phase. The estimated oxic growth rate in the presence of nitrite (linear regression of ln(V_e−O2_) against time was μ_ox_ = 0.11 (±0.02)h^−1^ (Figure 2B, Table 1A) and the estimated cell yield was only 14.1 (±1.1) cells pmol^−1^ e^−^ (Table 1A). The estimated V_max_ for oxygen respiration in cells grown in the presence of nitrite was 8.2 (±0.7) fmol e^−^ cell^−1^ h^−1^ (Table 1A). Thus, the presence of NO^−^_2_ in the medium appeared to exert an inhibitory effect on the oxygen respiration by terminal respiratory oxidases, resulting in lower V_max_ and cell yield per mol electron compared to cells grown in the presence of nitrate.

Finally, kinetics of O_2_ respiration were also analyzed when cells were incubated in vials containing minimal medium with 10 mM of NO^−^_3_, and an initial concentration of 5% N_2_O and 2% O_2_ in the headspace. Figure 3A shows the measured O_2_, NO, N_2_O, and N_2_ for a single vial throughout the 40 h incubation, as well as the OD_600_. In this case, oxygen was consumed within the first 15 h and the OD_600_ increased in proportion with the cumulative O_2_ consumption and continued increasing throughout the anoxic phase. Electron flow rate to O_2_ increased exponentially with an apparent growth rate (μ_ox_) = 0.28 (±0.03) h^−1^ (Figure 3B, Table 1A). Cell yield resulting from O_2_ respiration was very similar to that observed in nitrate-treated cells [23.1 (±6.2) cells pmol^−1^ e^−^ with a V_max_ of 8.9 (±0.13) fmol e^−^ cell^−1^ h^−1^] (Table 1A).

### Kinetics of NO^−^_3_ and NO^−^_2_ respiration

**Figure 4:**
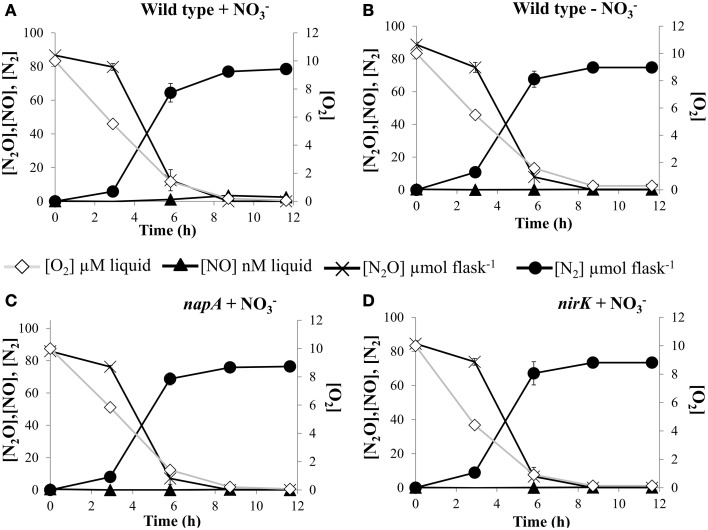
**NOx effect on kinetics of O_2_ and N_2_O depletion**. O_2_ and N_2_O consumption, and NO and N_2_ production by *E*. *meliloti* 1021 **(A,B)**, and *napA* **(C)** and *nirK* **(D)** mutant strains when incubated in the presence of 1% O_2_ plus 2% N_2_O in the headspace. Cells were incubated in minimal medium with **(A,C,D)** or without **(B)** 10 mM NO^−^_3_. Cultures with an initial OD_600_of 0.01 were vigorously stirred at 700 rpm. Plotted values are average of three replicate flasks for each treatment, with standard deviation (SD) as vertical bars (*n* = 3).

**Figure 5:**
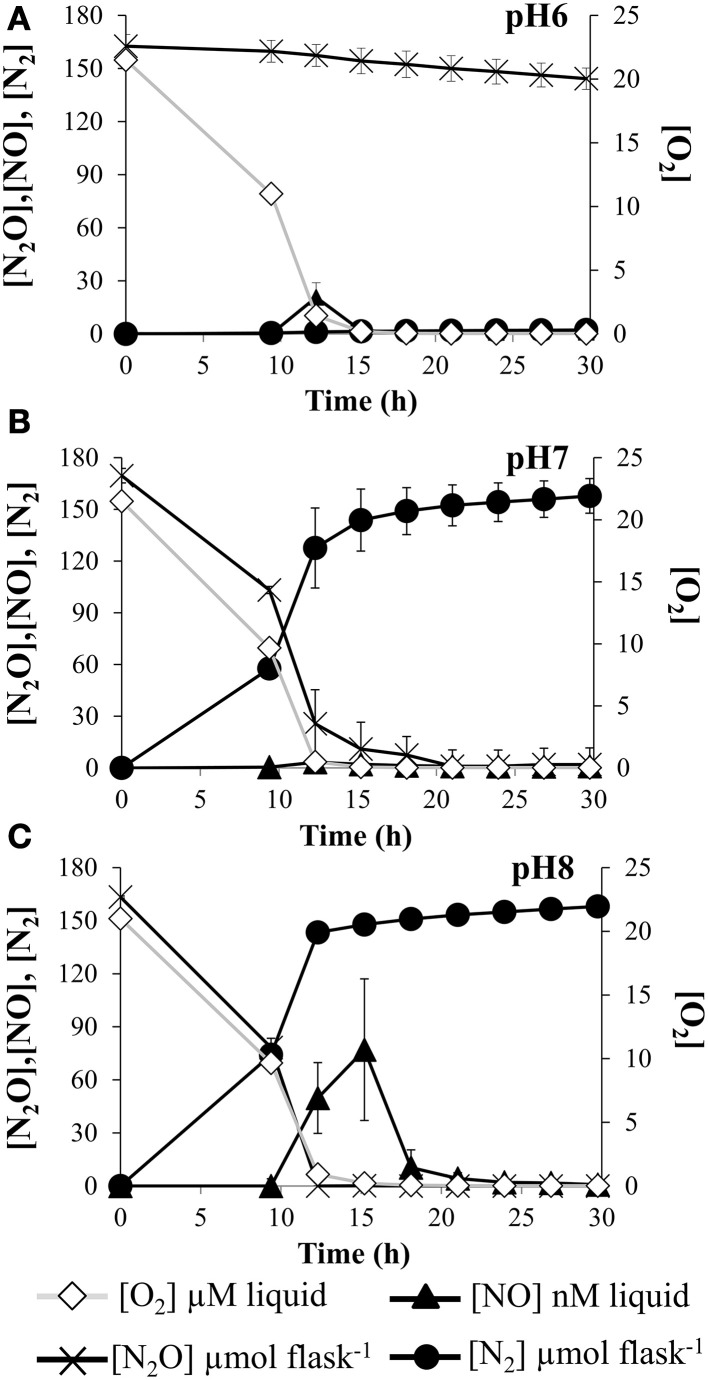
**pH effect on kinetics of O_2_ and N_2_O depletion**. O_2_ and N_2_O consumption, and NO and N_2_production by *E*. *meliloti* 1021 when incubated in the presence of 10 mM NO^−^_3_ in minimal medium at pH 6 **(A)**, pH 7 **(B)** and 8 **(C)**, and an initial O_2_ concentration of 02% in the headspace plus 5% N_2_O. Cultures with an initial OD_600_of 0.01 were vigorously stirred at 700 rpm. Plotted values are average of three replicate flasks for each treatment, with standard deviation (SD) as vertical bars (*n* = 3). The decline in N_2_O concentration at pH = 6 is due to sampling loss, not biological reduction of N_2_O to N_2_.

**Figure 6:**
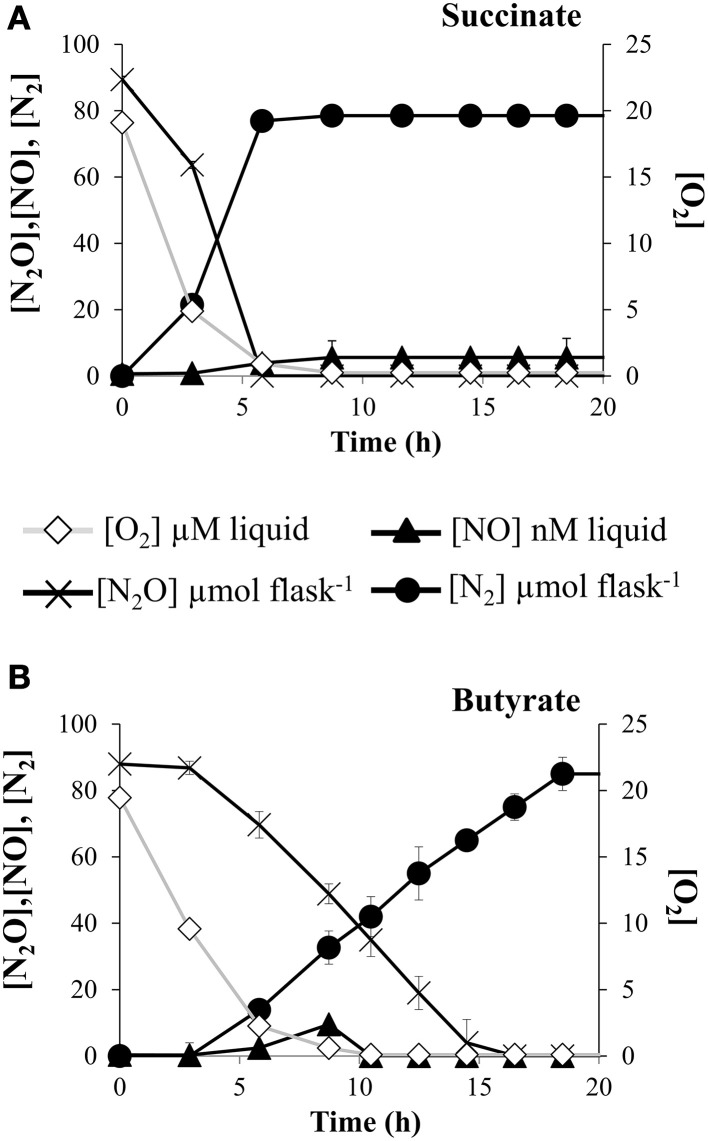
**C-source effect on kinetics of O_2_ and N_2_O depletion**. O_2_ and N_2_O consumption, and NO and N_2_ production by *E. meliloti* 1021 when incubated in the presence of 1% O_2_ plus 2 % N_2_O in the headspace, and 10 mM NO^−^_3_ in Robertsen's medium which contained succinate **(A)** or butyrate **(B)** as carbon sources. Cultures with an initial OD_600_ of 0.01 were vigorously stirred at 700 rpm. Plotted values are averages of three replicate flasks for each treatment, with standard deviation (SD) as vertical bars (*n* = 3).

When cells were cultured with NO^−^_3_, there was a very low NO^−^_3_ consumption rate as well as very low progressive accumulation of NO^−^_2_ throughout the entire anoxic phase (Figure 1A, insert), reaching only ~50 μmol vial^−1^ (which accounts for 10% of the NO^−^_3_-N in the medium). Very low levels of NO were also observed (12.40 ± 2.10 nM) after 40 h incubation (Table 1B, Figure 1A). Production of N_2_O in the headspace was insignificant and the fraction of NO^−^_3_ reduced to N_2_ at the end of the incubation was also extremely low (0.9 ± 0.3 %) (Table 1B, Figure 1A). When NO^−^_2_ was used as final electron acceptor, the first detection of NO occurred as the oxygen concentration in the liquid reached ~3 μM (Figure 2A, Table 1B). During the subsequent anoxic phase, NO continued to accumulate, reaching 94.20 ±16.90 nM levels at the end of the incubation period (Table 1B, Figure 2A). Similarly as for nitrate-treated cells (Figure 1A), production of N_2_O was undetectable and the total, cumulative production of N_2_ from the initially provided NO^−^_2_-N was also very low (0.18 ± 0.02 %) (Figure 2A, Table 1B). These data show that *E. meliloti* 1021 was clearly unable to shift effectively to NO^−^_3_ or NO^−^_2_ based anaerobic respiration. This inability was also confirmed by the lack of increase in measured OD_600_ throughout the anoxic phase (Figures 1, 2). Thus, the apparent growth rate during either NO^−^_3_ or NO^−^_2_ anoxic respiration (μ_anox_) was zero (Figures 1B, 2B, Table 1A). Similar growth rates were observed by using 1 mM or 500 μM NO^−^_2_ as electron acceptor (data not shown). One possible explanation to the lack of efficient reduction of NO^−^_3_ and NO^−^_2_ could be that rapid depletion of the oxygen in these cultures may have resulted in entrapment of the bacteria in anoxia, as shown previously for *P. denitrificans* by Bergaust et al. (2010). To test this hypothesis, we performed a follow-up experiment where the stirring speed was reduced from 700 rpm (used in the experiments reported in Figures 1, 2) to 200 rpm, in order to secure a slow transition from oxic to anoxic conditions in the liquid. These cultures showed the same lack of effective transition to denitrification as cultures with vigorous stirring, despite the fact that the cells with low stirring experienced a progressive O_2_ limitation during 50 h prior to complete O_2_ depletion (see Supplementary Figure S1).

### Kinetics of N_2_O respiration

The capacity of *E. meliloti* 1021 to reduce N_2_O was examined in vials containing 10 mM NO^−^_3_ in the medium plus 5% N_2_O and 2% O_2_ initially added to the headspace (Figure 3). As shown in Figure 3A, N_2_O was consumed rapidly and N_2_ production followed stoichiometrically the reduction of N_2_O to its complete depletion (100% of N_2_O was converted to N_2_ gas) (Figure 3A, Table 1B). As shown in Figure 3A, N_2_O reduction was at first detected at an O_2_ concentration of 5.9 (±2.6) μM (Table 1B). Traces of NO from NO^−^_3_ reduction were also detected (15 ±1.1 nM in the liquid; Table 1B). Final OD_600_ of cells incubated with N_2_O was clearly higher than that obtained when cells were incubated only with NO^−^_3_ or NO^−^_2_ as alternative electron acceptors (Table 1B), demonstrating the capacity of *E. meliloti* to couple N_2_O reduction with growth.

Electron flow to N_2_O increased with an apparent growth rate (μ_anox_) of 0.11 (±0.03) h^−1^ estimated by linear regression of ln (V_e−N2O_)against time (Figure 3B, Table 1A). Although low rates of electron flow to N_2_O occurred after 3 h, it increased sharply after 7 h as the electron flow to oxygen decreased due to oxygen depletion. Thus, the cells were evidently able to shift gradually from respiring O_2_ to N_2_O, preserving the total electron flow rate essentially unaffected after the depletion of oxygen. As shown in Table 1A, the estimated cell yield from N_2_O reduction was 18 (±0.6) cells pmole^−1^ e^−^. Knowing the yield in cell number per hour and the electron flow rate per hour we could estimate the V_max_ for N_2_O reduction to 5.7 (±1.1) fmol e^−^ cell^−1^ h^−1^ (Table 1A).

### No_x_ molecules do not trigger N_2_OR activity in *E. meliloti*

To evaluate the effect of NOx molecules as inducers of N_2_OR activity, we measured N_2_O uptake rates in cultures of *E. meliloti* 1021 strain that had received 10 mM NO^−^_3_ in the medium and compared this with cultures that were not supplemented with NO^−^_3_ (Figures 4A,B). The results showed similar N_2_O consumption as well as N_2_ production rates for the two treatments. Furthermore, no differences in N_2_O respiration was found between wild-type cells and strains which were defective in the *napA* and *nirK* structural genes when cultured in a medium amended with 10 mM NO^−^_3_ (Figures 4A,C,D). The *E. meliloti napA* or *nirK* mutants were demonstrated previously to be unable to reduce nitrate and nitrite respectively, to any further NOx intermediary of the denitrification process (Torres et al., 2014). These results suggested that the ability to reduce N_2_O was not affected by the presence or absence of NO, NO^−^_2_, or NO^−^_3_.

### Low pH severely impaires N_2_O uptake in *E. meliloti*

Since pH emerges as a master variable controlling the expression of N_2_O reductase, in this work we examined the pH effect on the kinetics of N_2_O reduction. For that purpose, *E. meliloti* cells were incubated in minimal medium strongly buffered with phosphate buffer, at pH 6, 7, and 8. Firstly, we grew *E. meliloti* 1021 cells aerobically to exponential (log) phase at pH 7. Then cells were transferred to the experimental vials containing 5% N_2_O and 2% O_2_ in the headspace and 10 mM NO^−^_3_ in the medium. Rates of O_2_ consumption were monitored until depletion and no differences were found between treatments. However, N_2_O reduction to N_2_ was completely blocked at pH 6 (Figure 5A). Surprisingly, when cells were incubated at pH 8, a significant peak of NO was detected. A negative effect of high pHs on *nor* expression or Nor activity could explain that transient peak of NO.

### Reduced C-sources attenuates N_2_O uptake in *E. meliloti*

Carbon availability is another key environmental factor affecting N_2_O production in the field. However, information about the implication of specific forms of reductants in N_2_O reductase activity is limited. Redox state of the C-sources might influence the amount of electrons available to reduce N_2_O to N_2_. For that reason, we tested the capacity of *E. meliloti* 1021 to reduce N_2_O in the presence of C-substrates with different redox potential, from highly oxidized as succinate or highly reduced such as butyrate. Aerobically raised cells were collected and inoculated into experimental vials containing minimal medium where glycerol was substituted by either succinate or butyrate. By using the robotized incubation system, rates of O_2_ respiration occurring previously to N_2_O consumption were also estimated. We found that O_2_ respiration from cells incubated in the presence of butyrate was slightly decreased when compared to cells incubated in the presence of succinate (Figures 6A,B). However, rates of N_2_O consumption were largely dependent on the oxidized or reduced nature of the carbon source. Thus, when butyrate was used as electron donor, the N_2_O reduction to N_2_ decreased about 3-fold compared to when succinate was used as the sole carbon substrate (Figures 6A,B).

## Discussion

In this work, we have used a robotized incubation system designed to simultaneously monitor with high sensitivity real-time changes in concentrations of O_2_, NO^−^_3_, NO^−^_2_ NO, N_2_O, and N_2_ during the transition from oxic to anoxic respiration. By using this system, we found that *E. meliloti* 1021 is unable to reduce NO^−^_3_ or NO^−^_2_ to N_2_O or N_2_ during the transition from oxic to anoxic conditions. Consequently, this bacterium was unable to sustain growth during anoxic conditions by using NO^−^_3_ or NO^−^_2_ as electron acceptors. This is in contrast to recent studies where growth of *E. meliloti* 1021 was observed during respiration of NO^−^_3_ as well as NO^−^_2_ (Torres et al., 2011a, 2014). This apparent discrepancy could be due to the different growth conditions and methodological approaches used by Torres et al. (2011a, 2014) and in this work. While they inoculated experimental vials with very high cell density (OD_600_~ 0.2–0.25), which were shaken at 170 rpm, the initial cell density used in the present work was significantly lower (OD_600_~ 0.01), and cultures were stirred at 700 rpm. The reason why we used different conditions in this work is to allow an efficient and controlled gas transfer from the headspace to the liquid and prevented cell aggregation and generation of localized micro-oxic spells during the aerobic phase previous to the transition to anaerobic respiration, as well as accumulation of toxic concentration of metabolites resulting from cell respiration. It might be possible that the growth conditions used by Torres et al. (2011a, 2014) provoked generation of anoxic micro-zones preceding total oxygen depletion due to cell aggregation and consequently the induction of *E. meliloti* 1021 denitrifying machinery would be facilitated. The present work extends the study of denitrification in *E. meliloti* by performing an estimation of the growth parameters (i.e., μ, yield, Vmax), as well as a precise quantification of NOx gases dynamics during the transition from oxic to anoxic respiration. This approach, never used in rhizobia, allowed us to perform an accurate estimation of the efficiency of the denitrifying process, and is regarded to be more physiologically relevant than previously conducted growth experiments.

When N_2_O was provided as an alternative electron acceptor, anaerobic respiration, and growth was sustained by reducing N_2_O to N_2_. In this context, a recent report showed the ability of *B. japonicum* USDA110 to grow anaerobically using exogenous N_2_O as the sole electron acceptor (Sanchez et al., 2013). Growth with N_2_O as electron acceptor has also been observed in *Anaeromyxobacter* (Sanford et al., 2012), and in *Wolinella, Campylobacter*, and *Geobacillus* (Liu et al., 2008; Kern and Simon, 2009) indicating that the atypical *nosZ* encodes a functional respiratory terminal N_2_O reductase in those bacteria. This is unlike *Pseudomonas aeruginosa* PAO1, which cannot grow on exogenous N_2_O as the only electron acceptor (Bryan et al., 1985; Zumft and Kroneck, 2007).

It is generally considered that low oxygen concentration is a requirement for expression of the denitrification machinery (van Spanning et al., 2007). Especially the N_2_OR has been considered as a very O_2_ labile reductase which is inactivated by the presence of low amounts of O_2_ (Alefounder and Ferguson, 1982; Coyle et al., 1985; Snyder and Hollocher, 1987). In contrast to these observations, our results suggest that expression of N_2_OR in *E. meliloti* might be subjected to a different regulation, in which N_2_O reduction occurs even in the presence of oxygen concentrations above 8 μM (Figure 3A).

It has been reported that expression and fine-tuning of the denitrification system also requires the presence of key molecules such as NO^−^_3_, NO^−^_2_, and NO which, through transcriptional factors and their protein-coupled sensory receptors, act as signals that trigger induction of the denitrification pathway (Zumft and Kroneck, 2007; Spiro, 2012). Our results suggested that oxygen limitation was the only prerequisite for maximal expression of N_2_OR in *E. meliloti*, although we cannot exclude that N_2_O is also necessary. The presence of a NOx (NO, NO^−^_2_, NO^−^_3_) was however not required, since N_2_OR activity remained at similar levels in the absence or in the presence of NO^−^_3_ in wild-type cells. Furthermore, in cells cultured with NO^−^_3_, no differences in N_2_OR activity were observed between wild-type, and the *napA* or *nirK* mutant strains where the reduction of NO^−^_3_ or NO^−^_2_ is blocked, respectively. In fact, previous studies of gene expression proposed that limited oxygen tension alone resulted in induction of the expression of the whole *nos* operon in *E. meliloti* (Bobik et al., 2006). In contrast to these findings, transcriptional profile analysis suggested that induction of *nosR* and *nosZ* gene expression also requires the presence of nitric oxide (Meilhoc et al., 2010). In line with this, recent studies using qRT-PCR showed that maximal transcription of the *E. meliloti nosZ* gene occurred when cells were subjected to anoxic conditions in the presence of nitrate (Torres et al., 2014). Similarly to our observations, it was recently reported that *P. denitrificans* is fully able to reduce N_2_O in the absence of oxyanions and NO (Bergaust et al., 2012). In contrast, it was proposed that the inability of *Pseudomonas aeruginosa* PAO1 and *Bacillus vireti* to grow on exogenous N_2_O as the only electron acceptor was because these organisms need NO as an inducer of *nosZ* transcription (Arai et al., 2003).

Our results clearly showed that *E. meliloti* 1021 was unable to express N_2_OR activity at pH 6. This difficulty in expressing N_2_OR at low pH was observed in *P. denitrificans* (Bergaust et al., 2010) and in suspensions of extracted soil bacteria (Liu et al., 2014). The phenomenon is ecologically important since there is ample evidence that low soil pH results in high N_2_O/N_2_ product ratios of denitrification (Raut et al., 2012; Qu et al., 2014).

Among the environmental factors that influence N_2_O emissions, and specifically the bacterial N_2_OR performance, very little is known about the mode in which availability and redox state of C-sources contribute. In this work, the observed attenuated N_2_OR activity in the presence of highly reduced C-sources could be attributed to a reduced capacity of cells to metabolize more complex C-substrates such as butyrate, causing a lowered electron flow through the respiratory chain, resulting in a reduced electron availability to reduce N_2_O to N_2_ by the N_2_OR (Morley and Baggs, 2011). Alternatively, a reduced efficiency to metabolize butyrate could be due to the fact that its uptake into cell probably requires active transport, and consequently cells may be subjected to periods of reduced N_2_OR activity (Schalk-otte et al., 2000). Supporting this hypothesis, it was found that N_2_OR activity was stimulated in the presence of artificial root exudates with easily metabolized C-sources such as glucose, as well as in soils amended with carbohydrates as glucose and starch (Murray et al., 2004; Henry et al., 2008). In addition, a regulatory control on *nos* transcription could also explain the dependence of the N_2_OR activity on the redox state of C-sources. In accordance with this, it was recently reported that expression levels of the *B. japonicum* NorC component of the nitric oxide reductase in wild-type cells, incubated in minimal medium with succinate as the sole C-source, were significantly higher than those observed in cells incubated in the presence of butyrate (Torres et al., 2011b). Similarly, expression of the *B. japonicum fixNOQP* genes, encoding the high affinity terminal oxidase *cbb*_3_, decreased when butyrate was the sole carbon source compared to when malate was used (Bueno et al., 2009).

Taken together, these results showed a novel denitrifying phenotype in *E. meliloti* 1021, for which the reduction of NO^−^_3_, or NO^−^_2_ was severely impaired, while N_2_O was actively reduced. We further demonstrated that the reduction of N_2_O sustained growth by *E. meliloti* 1021. To our knowledge this is the first time that it was demonstrated the capacity of *E. meliloti* to sustain anoxic respiration by using N_2_O as terminal electron acceptor. Since the effect of pH or C-sources on N_2_O reductase activity has never been examined in rhizobia, the relevance of this study is to demonstrate that both environmental factors affect N_2_O reductase activity in the model alfalfa endosymbiont, *E. meliloti* 1021. Although this strain is a model organism and is not commercially used as inoculant for alfalfa, the results obtained here could be expanded to more competitive and efficient N_2_-fixers inoculants in order to develop strategies to reduce N_2_O emissions from alfalfa crops. In fact, despite the large research efforts invested in flux measurement of N_2_O emissions, progress in developing efficient mitigation options has hitherto been slow. An essential objective should be to understand the underlying mechanisms and factors that affect the regulation of N_2_O consumption and production, and consequently to improve the product stoichiometry of denitrification (N_2_O/N_2_O + N_2_) in terrestrial ecosystems.

### Conflict of interest statement

The authors declare that the research was conducted in the absence of any commercial or financial relationships that could be construed as a potential conflict of interest.

## Supplementary material

Supplementary Figure S1 **Kinetics of O_2_ depletion and N_2_O, NO, and N_2_ production. *E. meliloti* 1021 was incubated in the presence of 10 mM NO^−^_3_ in minimal medium and an initial O_2_ concentration of 2% in the headspace. Cultures with an initial OD_600_ of 0.01 were vigorously stirred at 200 rpm. Plotted values are averages of three replicate flasks for each treatment, with standard deviation (SD) as vertical bars (*n* = 3)**.
