# Supplementary figures and images for: Anoxic growth of Ensifer meliloti 1021 by N2O-reduction, a potential mitigation strategy
Source: Front Microbiol. 2015 May 27;6:537. doi: 10.3389/fmicb.2015.00537 (PMC4443521; doi:10.3389/fmicb.2015.00537)

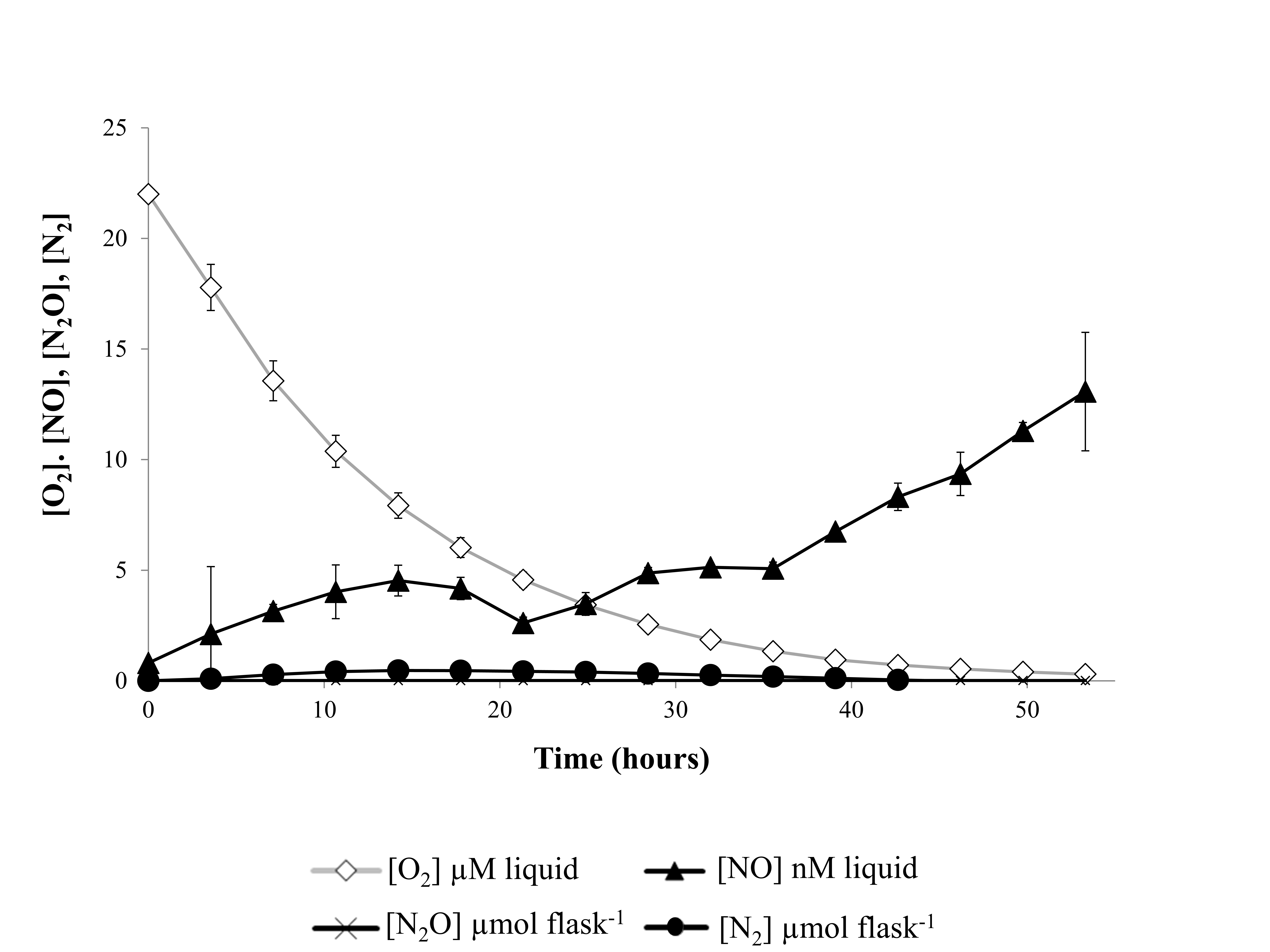

Supplement: Supplementary Figure S1 — Kinetics of O2 depletion and N2O, NO, and N2 production. E. meliloti 1021 was incubated in the presence of 10 mM NO−3 in minimal medium and an initial O2 concentration of 2% in the headspace. Cultures with an initial OD600 of 0.01 were vigorously stirred at 200 rpm. Plotted values are averages of three replicate flasks for each treatment, with standard deviation (SD) as vertical bars (n = 3). [file Image1.TIF]
